# LOX and LOXL2 Expression in Canine Mammary Carcinomas

**DOI:** 10.1111/vco.70036

**Published:** 2025-12-17

**Authors:** Jessika Daniel, Ingrid Kester Lima Silva, Rosemeri de Oliveira Vasconcelos, Adilson Paulo Marchioni Cabral, Andrigo Barboza De Nardi, Juliano Rodrigues Sangalli, Ricardo De Francisco Strefezzi

**Affiliations:** ^1^ Department of Veterinary Medicine, Laboratory of Comparative and Translational Oncology (LOCT‐USP), School of Animal Science and Food Engineering University of São Paulo (FZEA‐USP) Pirassununga São Paulo Brazil; ^2^ Veterinary Pathology Service, Department of Pathology, Reproduction and One Health, Faculty of Agricultural and Veterinary Sciences São Paulo State University (FCAV‐UNESP) Jaboticabal São Paulo Brazil; ^3^ Veterinary Oncology Service (SOV), Faculty of Agricultural and Veterinary Sciences São Paulo State University (FCAV‐UNESP) Jaboticabal São Paulo Brazil; ^4^ Department of Veterinary Medicine, School of Animal Science and Food Engineering University of São Paulo Pirassununga São Paulo Brazil

**Keywords:** cancer, carcinoma, dog, extracellular matrix, mammary gland, prognosis

## Abstract

Mammary tumours account for approximately 50% of the neoplasms in female dogs. Even conventionally accepted prognostic indicators often fail to reliably predict the clinical behaviour of these tumours, underscoring the need for more effective prognostic markers. Proteins of the LOX family are associated with tumour invasion and metastasis in several types of tumours. The purpose of this study was to characterise the immunohistochemical expression of LOX and LOXL2 in canine mammary carcinomas and to investigate their prognostic significance. Samples of mammary carcinomas from 80 female dogs with a minimum post‐surgical follow‐up of 180 days were analysed. Tumour samples were submitted to immunohistochemistry to detect LOX and LOXL2. Immunolabelling was evaluated based on scores for staining intensity and percentage of positive cells, and a combined score was used to classify each protein as having either ‘low‐’ or ‘high‐expression’. The results were compared with histological types, mortality due to the disease and post‐surgical survival. We found that negativity for LOXL2 expression was an indicator of higher risk of death due to the disease. Our results suggest that lysyl oxidases such as LOXL2 are potential prognostic markers in mammary carcinomas of dogs.

## 1 Introduction

Mammary tumours are the most common type of neoplasm in unspayed female dogs, being approximately 40%–50% malignant [1], particularly carcinomas [2]. The incidence rates vary by geographic region, tending to be lower in countries with higher rates of early spaying. The involvement of regional lymph nodes and the presence of metastases are reported as markers of poor prognosis [3].

In female dogs, even the most used prognostic indices, such as lymph node involvement and histological grade, have not shown a strong correlation with clinical behaviour. Assessing the expression of one or more immunomarkers, such as Ki67 index, COX‐2 and hormonal receptors, has proven to be a more reliable and conclusive tool [3, 4, 5].

The extracellular matrix (ECM) is a complex three‐dimensional network of macromolecules, composed of collagen, proteoglycans, glycosaminoglycans, elastin, fibronectin, laminins and other glycoproteins [6]. Collagen and elastin are its main constituents, becoming insoluble and stable through the action of the lysyl oxidase (LOX) enzyme family [7, 8]. This family consists of LOX and four LOX‐like enzymes, LOXL1 to LOXL4, which contribute to ECM remodelling by converting lysine and hydroxylysine residues in collagen and elastin—an essential process for enhancing tensile strength and maintaining the structural integrity of many tissues [9].

The LOXL2 protein contains an initial peptide and, on the N‐terminal portion, four cysteine‐rich scavenger receptor domains (SRCR) and a conserved LOX catalytic domain [10]. Janyasupab et al. [11] measured LOXL2 protein levels in serum, plasma and urine in humans and found that breast cancer patients had higher LOXL2 levels in the blood compared to healthy individuals. High expression of the LOX and LOXL2 enzymes is considered a risk factor for early metastasis in several types of cancer [12]. These enzymes can promote tumour cell migration and invasion, stimulate ECM remodelling and interact with several growth factors [13].

However, a paradoxical role for the LOX family members has been reported, associating low expression of these proteins with more invasive tumours. Zuber et al. [14] reported an approximately 60‐fold decrease in LOXL2 expression in RAS‐transformed rat fibroblasts. Low LOXL2 expression was also observed in ovarian tumours compared with healthy tissues [15]. It is suggested that in the early stages of cancer development, LOX cross‐linking activity may restrict tumour growth by limiting the expansion of its microenvironment. However, in later stages, LOX proteins appear to contribute to cancer progression by remodelling the ECM and creating a more permissive environment for cancer cell invasion and dissemination [16, 17].

The objective of the present study was to characterise the immunohistochemical expression of LOX and LOXL2 in mammary tumour samples from female dogs for prognostic evaluation, comparing the results with histological types, tumour‐related mortality and post‐surgical survival.

## 2 Materials and Methods

### 2.1 Samples

Samples of mammary carcinomas from female dogs were obtained retrospectively from the Tumour Bank of the Laboratory of Comparative and Translational Oncology at the School of Animal Science and Food Engineering, University of São Paulo (FZEA‐USP), as well as from the histopathological archives of the Faculty of Agricultural and Veterinary Sciences at São Paulo State University (UNESP). This study was approved by the Animal Use Ethics Committee (CEUA‐FZEA, protocol #7498190221). Cell line validation statement: no cell line was used in this study.

All cases of mammary carcinomas included in the study were surgically treated with complete tumour excision aiming for cure. Full mastectomy or nodulectomy, depending on the extension of the tumours, was performed with wide margins. The minimum clinical follow‐up for the cases that were censored by survival analysis (i.e., deaths unrelated to the disease, incomplete clinical history and animals alive at the end of the study) was 180 days. Information was obtained from medical records, interviews with owners and veterinarians in charge, and follow‐up consultations.

### 2.2 Histopathology and Immunohistochemistry

The samples were processed using routine histology techniques and 4‐μm sections were stained with Haematoxylin & Eosin for confirmation of the diagnosis and histological grading of the lesions. Tumours were classified and graded by three simultaneous observers, following the proposal of Zappulli et al. [18], with the final diagnosis decided by consensus. In cases of multiple lesions with different histological subtypes, the most aggressive tumours were selected [19, 20].

For immunohistochemistry, the histological sections were placed on silanised slides (Starfrost) and deparaffinised. Heat induced antigen retrieval was performed in a steamer for 25 min at 95°C, with Citrate buffer pH 6.0. Non‐specific binding blocking was conducted with 5% skim milk solution (LOX) in a water bath (60°C) for 1 h, or with goat milk at room temperature for 15 min (LOXL2). Endogenous peroxidase blocking was carried out at room temperature for 1 h with a 3% H_2_O_2_ solution in a dark chamber. The slides were incubated with primary antibodies at 4°C in a humidified dark chamber overnight. The number of samples and primary antibodies are listed in Table 1. The secondary antibody used was EasyLink One (LSAB+System‐HRP, EasyPath) for 25 min at room temperature. Primary antibodies were selected based on the similarity between target epitopes and the respective canine protein through sequence alignment via BLAST. Additionally, reactivity was validated by western blotting in samples of canine mammary carcinoma, lung and mouse spleen [21, 22]. Positive controls were human and canine mammary carcinoma. Negative controls were performed by replacing the primary antibody with normal rabbit IgG at the same concentration. Reactions were visualised with 3,3′‐diaminobenzidine tetrahydrochloride hydrate chromogen (DAB, Erviegas) and Harris Haematoxylin was used for counterstaining.

**TABLE 1:** List of primary antibodies used, number of samples processed, concentration and positive controls for each protein in the study.

Protein	Antibody	*n*	Dilution
LOX	Rabbit polyclonal anti‐LOX (PA5‐27270, ThermoFisher)	51	1:200
LOXL2	Rabbit polyclonal anti‐LOXL2 (LS‐A9350‐50, LSBio, LifeSpan Biosciences)	56	1:500

The immunohistochemical labelling was quantified in five non‐overlapping images from random fields: the first in the central area of the tumour section and the remaining four around the first at every 90°. The images were taken from intratumoral areas, avoiding necrotic regions, on a microscope (Leica DM500) attached to a high‐resolution digital camera (ICC50HD, Leica) using the ×40 objective (each image area = 0.08 mm^2^). The parameters evaluated were: presence or absence of staining in epithelial cells, staining intensity scores (0 = negative; 1 = weak; 2 = strong) and scores for the percentage of positive epithelial cells (0 = negative; 1 = 0%–25%; 2 = 25%–50%; 3 = 50%–75%; 4 = 75%–100%). A combined score was obtained through summing of intensity and percentage scores (total of 0–3 = low expression; 4–6 = high expression), according to Strefezzi et al. [23]. The subcellular localisation (cytoplasmic and/or nuclear) was evaluated in epithelial cells. Myoepithelial cells and fibroblasts were classified as positive or negative in each tumour sample.

### 2.3 Statistical Analysis

The data obtained were compared using the Mann–Whitney or *χ* ^2^ tests, depending on the type of data analysed. Post‐surgical survival was analysed by the Kaplan–Meier method followed by Mantel‐Cox (log‐rank) or Gehan–Breslow–Wilcoxon tests. Correlations between parameters were assessed with Spearman's test. Due to the variety of histological types and the limited sample size for certain subtypes, cases were divided into three groups: G1, or ‘Low malignancy’ (mixed carcinoma and complex carcinoma); G2, or ‘Moderate malignancy’ (tubular, tubulopapillary, intraductal papillary and ductal carcinomas and carcinoma and malignant myoepithelioma) and G3, or ‘High malignancy’ (solid, anaplastic, micropapillary and comedocarcinomas) [19].

Statistical calculations were performed using GraphPad Prism software (version 9.5.1 for MacOS, GraphPad Software Inc.) and the significance level was set at 5%.

## 3 Results

Eighty samples of canine mammary carcinomas from 69 dogs were selected for immunohistochemical evaluation. The most frequent histological subtype was complex carcinoma (26/80, 32.5%), followed by tubular carcinoma (20/80, 25%; Table 2).

**TABLE 2:** Distribution of mammary carcinomas according to histological subtype.

Histological type	Number of cases	%
Complex carcinoma	26	32.50
Tubular carcinoma	20	25.00
Tubulopapillary carcinoma	13	16.25
Mixed carcinoma	8	10.00
Intraductal papillary carcinoma	7	8.75
Carcinoma and malignant myoepithelioma	2	2.50
Comedocarcinoma	2	2.50
Micropapillary carcinoma	1	1.25
Solid carcinoma	1	1.25
Total	80	100.0

The average age at diagnosis was 10.5 years, ranging from 5 to 17 years. Most animals were of mixed breed (20/69, 28.9%), followed by Pinscher (8/69, 11.59%), Dachshund (7/69, 10.14%) and Poodle (4/69, 5.69%). Other breeds represented in the study included Brazilian terrier, Yorkshire terrier, Shih Tzu, Border Collie, Golden Retriever, Labrador Retriever, Lhasa Apso, Cocker Spaniel, Boxer, Doberman pinscher, Maltese and German Shepherd. Fifty (72.4%) of the dogs were neutered, three were intact at the time of surgery (4.4%), and in 16 cases (23.2%), this information was not available in the clinical records.

**FIGURE 1:**
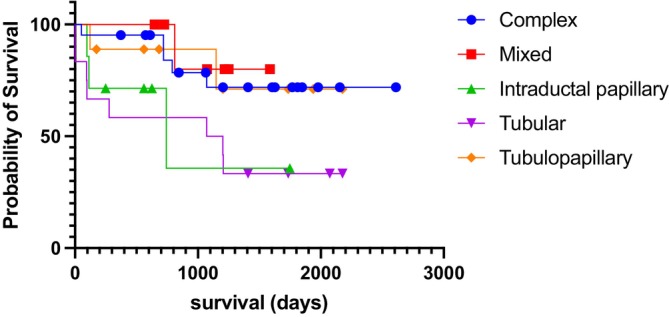
Post‐surgical survival curves in function of the histological subtypes of mammary carcinomas (*χ* ^2^ = 9.606; *p* = 0.0476; median survival = 742 days for Intraductal papillary and 1136 days for Tubular carcinoma; Kaplan–Meier followed by Gehan–Breslow–Wilcoxon test; *χ* ^2^ = 4.790; *p* = 0.0286; hazard ratio = 3.219 between tubular and complex types; Kaplan–Meier followed by Mantel‐Cox/log‐rank test).

In 28.9% (20/69) of cases, death was attributed to the tumour, and in 27.53% (19/69), dogs died from unrelated causes. The cause of death was unknown in 12.5% (7/69) of cases, and at the end of the study, 28.98% (20/69) of the patients were still alive. A survival analysis in function of the histological types was conducted on subtypes with more than 6 available cases, regardless of histological grades. Statistically significant differences were detected among tumour types (*χ* ^2^ = 9.606; *p* = 0.0476; median survival = 742 days for Intraductal Papillary Carcinoma and 1136 days for Tubular Carcinoma; Figure 1), especially between tubular and complex types (*χ* ^2^ = 4.790; *p* = 0.0286; hazard ratio = 3.219).

**FIGURE 2:**
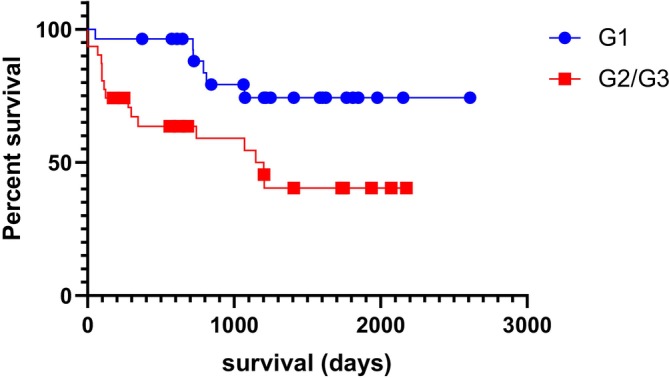
Post‐surgical survival curves for dogs with low malignancy (G1) and moderate to high malignancy carcinomas (G2/G3) (*χ* ^2^ = 6.702, *p* = 0.0096, hazard ratio = 3.200; median survival for G2/G3 = 1148 days; Kaplan–Meier followed by Mantel‐Cox/log‐rank test).

Since G3 (high malignancy tumours) consisted of only four cases, we chose to analyse them grouped with G2 cases. Dogs in the G2/G3 group had a shorter post‐surgical survival (*χ* ^2^ = 6.702, *p* = 0.0096, median survival = 1148 days; Figure 2).

**FIGURE 3:**
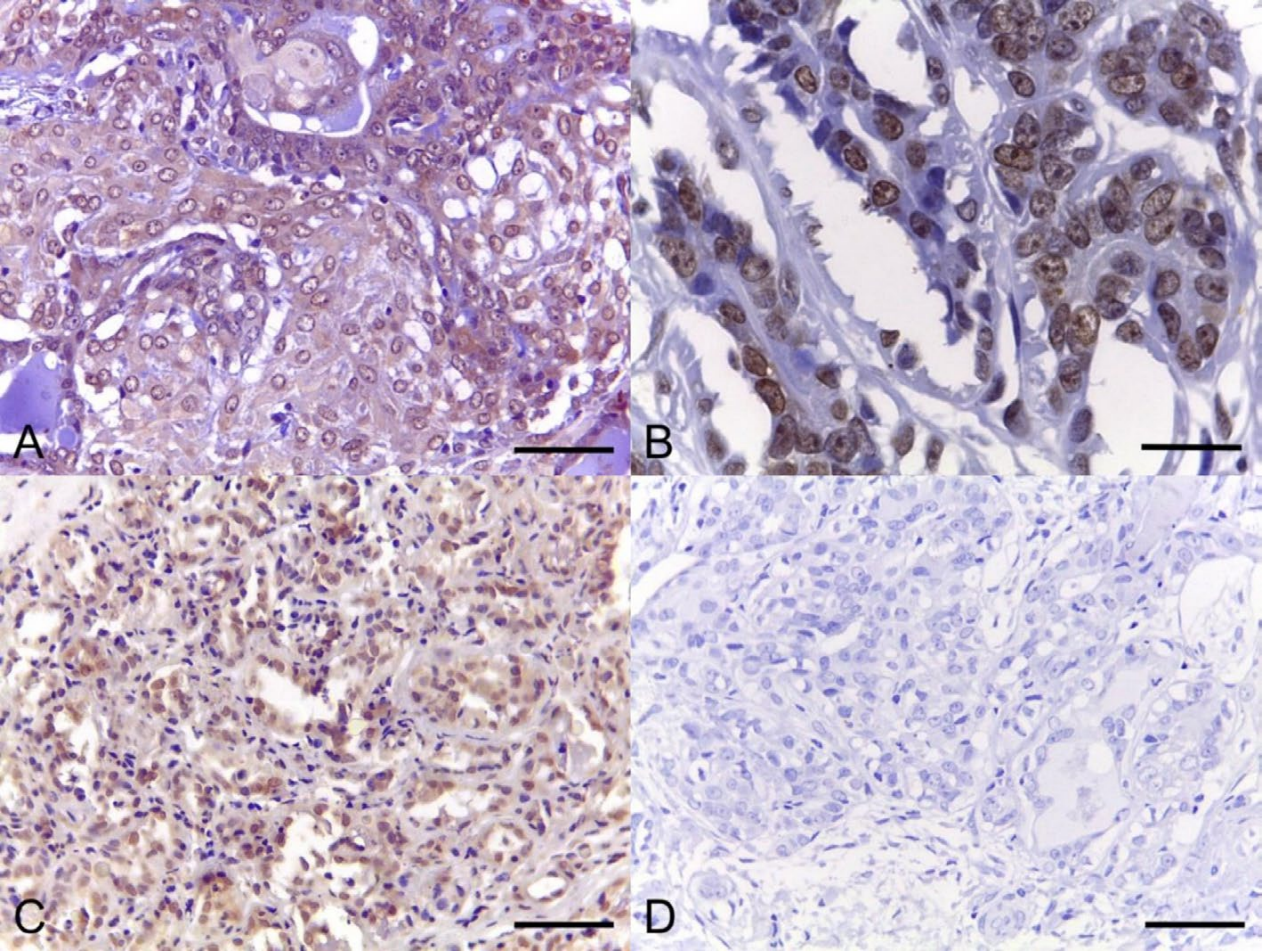
Photomicrographs of canine mammary carcinomas showing immunostaining for LOX: (A) strong cytoplasmic staining in a complex carcinoma (bar = 50 μm); (B) nuclear staining (bar = 20 μm); and (C) nuclear and cytoplasmic staining (bar = 50 μm) and (D) negative control in a tubulopapillary carcinoma (bar = 50 μm). Immunohistochemistry, DAB, counterstained with Harris' haematoxylin.

**FIGURE 4:**
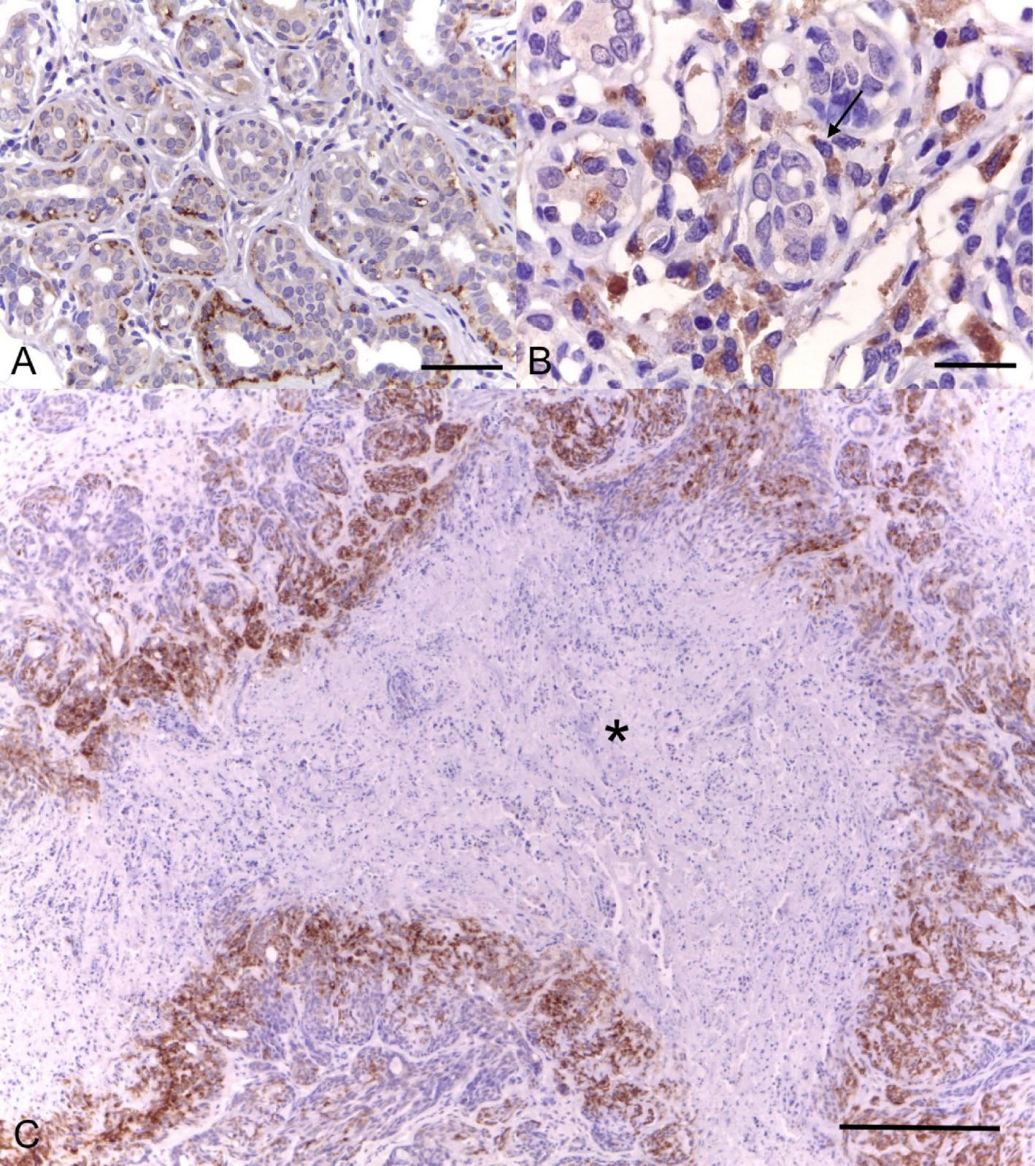
Photomicrographs of canine mammary tubular carcinomas showing immunostaining for LOXL2: (A and B) weak cytoplasmic staining in epithelial cells and strong cytoplasmic staining in myoepithelial cells (arrow), with a finely granular pattern (bars = 50 and 20 μm, respectively) and (C) positive cells surrounding a necrotic area (asterisk; bar = 200 μm). Immunohistochemistry, DAB, counterstained with Harris' haematoxylin.

The LOX protein (Figure 3) was found in the cytoplasm of the neoplastic epithelial cells in 78.4% of cases (40/51) and in both the nucleus and cytoplasm in 11.8% (6/51). Three cases showed both ECM and cytoplasm staining, and 2 cases were negative for LOX. Ninety‐six percent of the cases presented high LOX expression. LOX was also found in the cytoplasm of myoepithelial cells in 74% of the cases. The LOXL2 protein (Figure 4) was detected in the cytoplasm of the neoplastic cells in 69.64% (39/56) of cases, in both the ECM and cytoplasm in 12.50% (7/56), in the nucleus and cytoplasm in 7.14% (4/56), and 10.71% (6/56) of the cases were negative. Regarding the combined scores, 48.21% of the cases were classified as high LOXL2 expression and 51.78% as low LOXL2 expression. Myoepithelial cells were positive in 30.35% of the cases (Figure 4A). In all cases, the staining pattern was strong and finely granular.

All tumours analysed in the group of animals that were censored in the survival analysis were positive for LOXL2 in epithelial cells (29/29, 100%) versus 76.92% (10/13) of the tumours in animals that died due to the tumour (*p* = 0.0249). There was no difference between the G1 and G2/G3 cases (*p* = 0.6156) regarding LOXL2 scores. We did not detect differences regarding LOX expression between censored cases and those that died due to the tumour in terms of percentage (*p* = 0.8451) and intensity of staining (*p* = 0.3339) or final score (*p* = 0.8460).

No statistically significant differences were found for LOX and LOXL2 between the histological groups of high, moderate and low malignancy regarding the presence/absence of staining in epithelial cells, the percentage of positive epithelial cells and the staining intensity (both individually and as a summed score), as well as the presence/absence of staining in myoepithelial cells and fibroblasts (Table 3). No significant differences were detected in survival when comparing the high versus low LOXL2 scores in epithelial cells (*p* = 0.6217), and the histological grades (*p* = 0.2437).

**TABLE 3:** *p* values for the comparisons between low‐malignancy (G1) and intermediate/high‐malignancy (G2/G3) histological groups based on the presence or absence of staining in epithelial cells, percentage and intensity of staining and presence/absence of staining in myoepithelial cells and fibroblasts.

	Epithelial cells positivity^a^	Epithelial cells percentage score^b^	Epithelial cells intensity score^b^	Epithelial cells combined score^b^	Myoepithelial cells positivity^a^	Fibroblasts positivity^a^
LOXL2	> 0.9999	0.9218	0.2697	0.6156	0.5785	> 0.9999
LOX	—	0.7837	0.7312	0.3301	0.0658	> 0.9999

^a^ Fisher's exact test.

^b^ Mann–Whitney test.

## 4 Discussion

In the present study, the expressions of LOX and LOXL2 were investigated in canine mammary carcinomas to verify their possible relationships with post‐surgical survival and tumour‐related mortality. We found that the absence of LOXL2 indicates a higher likelihood of death due to the disease.

LOXL2 responds to hypoxic conditions by stabilising HIF1A and generating hydrogen peroxide [24]. In our study, we observed a pattern of higher protein expression around tissue necrosis foci, probably due to hypoxia in these locations. Higher expressions of LOX and LOXL2 are generally associated with worse prognosis and higher malignancy in cancer patients [11, 12, 13]. These proteins act in the extracellular environment, facilitating ECM remodelling and promoting invasion and metastasis. We believe that the relationship between LOXL2 and the lower likelihood of death due to the tumour demonstrated by our data is due to a paradoxical effect, revealing that they can act as both tumour suppressors and metastasis promoters [16, 17]. These results may be due to their multiple temporal expression patterns. In early stages, LOX crosslinking activity may limit tumour growth by hindering the expansion of the tumour microenvironment, while the opposite happens in more advanced stages of the disease. This dual effect reflects the influence of the microenvironment and tissue oxygenation state on LOX function, emphasising its complex role in oncogenesis and cancer progression.

These findings offer new perspectives on the role of LOX and LOXL2 proteins in canine mammary neoplasms, challenging some previous perceptions about the relationship of these proteins with a worse prognosis and suggesting that they may have a more complex and potentially beneficial role than previously considered [17]. The present study was the first to evaluate the expression of these proteins together and through immunohistochemistry in canine mammary carcinoma samples, which allowed for better visualisation of the location of these proteins.

A limitation we encountered was the small number of samples in some of the histological subtypes. Therefore, we chose to group the cases according to malignancy to enable more consistent statistical analyses, including the maximum possible number of samples. Similar to previous studies [20, 25], our population was composed of a group of ‘low malignancy’ carcinomas, representing approximately 70% of the total cases, and only 30% of ‘high malignancy’ carcinomas, which may have reduced our ability to detect statistical differences. However, we also demonstrated that dogs with moderate/high malignancy carcinomas had a significantly shorter survival compared with those with low malignancy tumours. Despite being an expected result, these data also confirm the validity of the criteria used in our study for grouping the mammary carcinoma cases.

Tumour heterogeneity may also influence the expression of the proteins that were investigated. We believe that larger sample sizes would likely yield more representative results, thereby enabling comparisons of molecular expression with specific clinical outcomes within each histological subtype. A larger number of cases would also allow for the evaluation of the contribution of myoepithelial cells in mixed‐type or complex carcinomas, particularly as these cells are present in only a subset of mammary neoplasms.

An additional limitation of the present study is the lack of complete clinical information required for TNM staging in some cases—such as tumour size, lymph node status and presence of distant metastases—as these data were not systematically recorded for all cases analysed.

Future investigations targeting these issues may contribute to a better understanding of the biological relevance of these proteins in canine mammary carcinomas. Additionally, since all samples were obtained via excisional biopsy with curative intent, it was not possible to monitor the natural progression of the tumours over time, preventing a longitudinal analysis of cancer progression. Also, we recognise that the use of clinical samples might have introduced some bias into the results, due to variations in fixation time and antigen preservation, which can affect staining intensity. To minimise these variables, we used samples from University Veterinary Hospitals, where tissue sampling, fixation and processing of tissues are performed following a consistent and standardised protocol.

We believe that LOX and LOXL2 may play distinct roles in canine mammary tumorigenesis, tumour progression, angiogenesis and metastasis, and that future mechanistic studies are warranted to elucidate the molecular mechanisms underlying tumour behaviour.

## Funding

This work was supported by Fundação de Amparo à Pesquisa do Estado de São Paulo, 2020/10582‐0, 2020/12789‐1 and Conselho Nacional de Desenvolvimento Científico e Tecnológico, 303748/2021‐4.

## Conflicts of Interest

The authors declare no conflicts of interest.

## Supporting information


**Table S1:** Description of the samples regarding histologic type, histologic group, grade and scores for LOXL2 and LOX.

## Data Availability

The data that support the findings of this study are available from the corresponding author upon reasonable request.
