# Supplementary material for: LOX and LOXL2 Expression in Canine Mammary Carcinomas
Source: Vet Comp Oncol. 2025 Dec 17;24(1):160–6. doi: 10.1111/vco.70036 (PMC12875756; doi:10.1111/vco.70036)
Supplement: Supplementary file 1 — Table S1: Description of the samples regarding histologic type, histologic group, grade and scores for LOXL2 and LOX. [file VCO-24-160-s001.pdf]

Supplementary Table 1 – Description of the samples regarding histologic type, histologic group, grade, status, survival and scores for LOXL2 and LOX.

Animal ID	Lesion ID	Histologic type	Histo group	Grade	Status	survival (days)	LOXL2 score	LOX score
1	1	Complex carcinoma	1	II	1	1070	5	4
1	2	Tubular carcinoma	2	I	1	1070	n.d.	n.d.
2	1	Tubular carcinoma	2	II	1	1	3	6
2	2	Tubular carcinoma	2	I	1	1	5	6
3	1	Complex carcinoma	1	I	0	1848	2	6
3	2	Complex carcinoma	1	I	0	1848	3	5
4	1	Tubular carcinoma	2	II	0	22	n.d.	5
4	2	Tubular carcinoma	2	II	0	22	n.d.	n.d.
5	1	Complex carcinoma	1	I	n.d.	n.d.	4	n.d.
6	1	Complex carcinoma	1	II	0	1204	4	n.d.
6	2	Tubulopapillary carcinoma	2	n.d.	0	1204	5	5
7	1	Tubulopapillary carcinoma	2	II	n.d.	n.d.	5	5
8	1	Tubular carcinoma	2	n.d.	1	97	0	5
8	2	Intraductal papillary carcinoma	2	I	1	97	n.d.	5
9	1	Complex carcinoma	1	II	0	1408	5	n.d.
10	1	Tubulopapillary carcinoma	2	n.d.	0	2176	3	4
10	2	Tubular carcinoma	2	I	0	2176	4	n.d.
11	1	Tubular carcinoma	2	I	n.d.	n.d.	n.d.	n.d.
11	2	Tubular carcinoma	2	I	n.d.	n.d.	n.d.	n.d.
12	1	Tubulopapillary carcinoma	2	I	0	560	4	5
12	2	Intraductal papillary carcinoma	2	I	0	560	n.d.	n.d.
13	1	Tubulopapillary carcinoma	2	II	1	1148	2	5
14	1	Tubulopapillary carcinoma	2	n.d.	n.d.	n.d.	5	n.d.
14	2	Tubulopapillary carcinoma	2	I	n.d.	n.d.	5	5
15	1	Complex carcinoma	1	II	1	53	n.d.	5
16	1	Mixed carcinoma	1	I	0	48	n.d.	n.d.
17	1	Tubular carcinoma	2	n.d.	1	94	n.d.	n.d.
18	1	Complex carcinoma	1	I	n.d.	n.d.	3	n.d.
19	1	Carcinoma and malignant myoepitelioma	2	II	1	298	n.d.	5
20	1	Tubular carcinoma	2	II	0	1734	3	n.d.
20	2	Tubulopapillary carcinoma	2	II	0	1734	6	3
21	1	Intraductal papillary carcinoma	2	I	0	626	n.d.	5
22	1	Mixed carcinoma	1	I	0	648	n.d.	5
23	1	Complex carcinoma	1	II	0	844	0	5
24	1	Tubulopapillary carcinoma	2	I	1	122	4	0
25	1	Complex carcinoma	1	III	1	721	0	0
26	1	Intraductal papillary carcinoma	2	I	1	112	n.d.	n.d.
27	1	Complex carcinoma	1	II	0	1978	n.d.	n.d.
28	1	Comedocarcinoma	3	I	1	69	n.d.	6
29	1	Tubular carcinoma	2	II	n.d.	n.d.	3	5
30	1	Tubular carcinoma	2	II	0	99	4	5
31	1	Micropapillary carcinoma	3	n.d.	n.d.	n.d.	n.d.	5
32	1	Tubular carcinoma	2	II	1	277	4	n.d.
33	1	Tubulopapillary carcinoma	2	III	0	684	5	n.d.

34	1	Complex carcinoma	1	II	n.d.	n.d.	4	n.d.
35	1	Tubular carcinoma	2	III	n.d.	n.d.	3	5
36	1	Tubulopapillary carcinoma	2	III	n.d.	n.d.	n.d.	n.d.
37	1	Tubular carcinoma	2	I	n.d.	n.d.	3	5
38	1	Complex carcinoma	1	I	0	2611	3	n.d.
39	1	Complex carcinoma	1	I	0	2154	5	n.d.
40	1	Intraductal papillary carcinoma	2	I	1	742	n.d.	n.d.
41	1	Tubular carcinoma	2	I	0	2073	n.d.	n.d.
42	1	Complex carcinoma	1	I	0	1810	5	5
43	1	Complex carcinoma	1	I	1	719	2	6
44	1	Intraductal papillary carcinoma	2	I	0	249	3	6
45	1	Complex carcinoma	1	II	0	611	3	n.d.
46	1	Mixed carcinoma	1	I	0	1074	4	5
47	1	Complex carcinoma	1	I	0	1625	6	6
48	1	Complex carcinoma	1	I	0	574	2	5
49	1	Solid carcinoma	3	III	1	344	5	5
50	1	Complex carcinoma	1	II	0	1604	4	5
51	1	Complex carcinoma	1	I	0	61	0	6
52	1	Tubulopapillary carcinoma	2	II	0	1938	3	4
53	1	Complex carcinoma	1	I	1	790	5	6
54	1	Tubular carcinoma	2	II	0	1408	4	n.d.
55	1	Mixed carcinoma	1	I	0	1586	3	6
56	1	Complex carcinoma	1	I	0	1767	5	n.d.
57	1	Complex carcinoma	1	II	0	1064	3	n.d.
58	1	Carcinoma and malignant myoepitelioma	2	I	0	132	6	5
59	1	Tubular carcinoma	2	III	1	1206	0	5
60	1	Tubular carcinoma	2	II	1	1203	3	5
61	1	Mixed carcinoma	1	I	0	1249	4	5
62	1	Mixed carcinoma	1	I	0	725	n.d.	4
63	1	Complex carcinoma	1	I	0	372	4	6
64	1	Mixed carcinoma	1	I	0	1217	3	6
65	1	Comedocarcinoma	3	I	n.d.	n.d.	0	6
66	1	Complex carcinoma	1	n.d.	n.d.	n.d.	n.d.	5
67	1	Mixed carcinoma	1	I	1	810	n.d.	5
68	1	Intraductal papillary carcinoma	2	I	0	1747	n.d.	6
69	1	Tubulopapillary carcinoma	2	I	0	174	n.d.	n.d.

n.d. = not determined; Status: 0 = censored; 1 = death due to the disease.
